# Phase II study of necitumumab plus modified FOLFOX6 as first-line treatment in patients with locally advanced or metastatic colorectal cancer

**DOI:** 10.1038/bjc.2015.480

**Published:** 2016-01-14

**Authors:** E Elez, A Hendlisz, T Delaunoit, J Sastre, A Cervantes, R Varea, G Chao, J Wallin, J Tabernero

**Affiliations:** 1 Vall d'Hebron Institute of Oncology Barcelona, Spain and Universitat Autònoma de Barcelona, 08035 Barcelona, Spain; 2 Institut Jules Bordet, 1000 Brussels, Belgium; 3 Centres Hospitaliers Jolimont, 7100 Haine Saint-Paul, Belgium; 4 Hospital Universitario San Carlos, 28040 Madrid, Spain; 5 Biomedical Research Institute INCLIVA, University of Valencia, 46010 Valencia, Spain; 6 Eli Lilly and Company, 28108 Madrid, Spain; 7 Eli Lilly and Company, 08807 Bridgewater, NJ, USA; 8 Eli Lilly and Company, 16973 Stockholm, Sweden

**Keywords:** advanced colorectal cancer, modified FOLFOX6, necitumumab, KRAS, EGFR

## Abstract

**Background:**

This single-arm phase II study investigated the EGFR monoclonal antibody necitumumab plus modified FOLFOX6 (mFOLFOX6) in first-line treatment of locally advanced or metastatic colorectal cancer (mCRC).

**Methods:**

Patients received 800-mg intravenous necitumumab (day 1; 2-week cycles), followed by oxaliplatin 85 mg m^−2^, folinic acid 400 mg m^−2^, and 5-fluorouracil (400 mg m^−2^ bolus then 2400 mg m^−2^ over 46 h). Radiographic evaluation was performed every 8 weeks until progression. Primary endpoint was objective response rate.

**Results:**

Forty-four patients were enrolled and treated. Objective response rate was 63.6% (95% confidence interval 47.8–77.6); complete response was observed in four patients; median duration of response was 10.0 months (7.0–16.0). Median overall survival (OS) and progression-free survival (PFS) were 22.5 (11.0–30.0) and 10.0 months (7.0–12.0), respectively. Clinical outcome was better in patients with *KRAS* exon 2 wild type (median OS 30.0 months (23.0–NA); median PFS 12.0 (8.0–20.0)), compared with *KRAS* exon 2 mutant tumours (median OS 7.0 months (5.0–37.0); median PFS 7.0 (4.0–18.0)). The most common grade ⩾3 adverse events were neutropenia (29.5%), asthenia (27.3%), and rash (20.5%).

**Conclusion:**

First-line necitumumab+mFOLFOX6 was active with manageable toxicity in locally advanced or mCRC; additional evaluation of the impact of tumour *RAS* mutation status is warranted.

Colorectal cancer (CRC) is the third most common form of cancer in men and the second most common form of cancer in women worldwide (Ferlay *et al*, 2013). An estimated annual total of 1.4 million cases will be diagnosed, leading to ∼694 000 deaths per year (∼8.5% of all cancer-related deaths). Approximately, 25% of CRC cases are overtly metastatic at diagnosis, and ∼50% of patients will ultimately develop recurrent or metastatic disease (Van Cutsem *et al*, 2014).

Many phase II–III trials have investigated the addition of an epidermal growth factor receptor (EGFR) monoclonal antibody (mAb) to a first-line FOLFOX (oxaliplatin, folinic acid and 5-fluorouracil (5-FU)) combination in patients with KRAS exon 2 wild-type metastatic CRC (mCRC). The phase III PRIME study (Douillard *et al*, 2010, 2014) and the randomised phase II OPUS study (Bokemeyer *et al*, 2011) evaluated the FOLFOX4 regimen in combination with panitumumab and cetuximab, respectively. In these studies, objective response rate (ORR) and progression-free survival (PFS) were significantly improved with the addition of an EGFR mAb to FOLFOX4 among patients with tumours assessed as wild type at codons 12 and 13 of *KRAS* exon 2; in the PRIME study, a significant improvement in overall survival (OS) was also observed. Extended mutation analyses of additional tumour *RAS* loci (*KRAS* exons 3 and 4, and *NRAS* exons 2, 3 and 4) suggested that the efficacy benefit was further restricted to patients with tumours wild type at all screened loci (Douillard *et al*, 2013; Bokemeyer *et al*, 2015). In both the PRIME and OPUS studies, a negative effect on efficacy was reported from combining an EGFR mAb with FOLFOX4 among patients whose tumours harboured a *RAS* mutation (Douillard *et al*, 2010, 2013; Bokemeyer *et al*, 2011, 2015). On the basis of additional evidence showing anti-EGFR antibodies were unlikely to benefit patients with this disease whose tumours carry *KRAS* mutations (Amado *et al*, 2008; Karapetis *et al*, 2008), the phase III COIN trial evaluated the addition of cetuximab to oxaliplatin-based chemotherapy (oxaliplatin plus capecitabine or oxaliplatin plus fluorouracil and folinic acid) in first-line treatment of patients with advanced CRC with KRAS wild-type tumours (Maughan *et al*, 2011). In this trial, cetuximab increased the response rate; no evidence of benefit in PFS or OS was seen.

On the basis of these data, FOLFOX4 in combination with either panitumumab or cetuximab is recommended in Europe and the United States for the first-line treatment of patients with *RAS* wild-type mCRC only (NCCN).

Necitumumab (LY3012211; IMC-11F8) is a second-generation recombinant human EGFR mAb of the immunoglobulin G1 class, which demonstrates a high affinity for EGFR and blocks ligand-induced receptor phosphorylation and downstream signalling (Liu *et al*, 2004). *In vitro* studies further demonstrate that necitumumab inhibits EGFR-dependent tumour cell proliferation, and can exert cytotoxic effects in tumour cells through antibody-dependent cell-mediated cytotoxicity. Necitumumab has also been shown to block tumour growth in CRC xenograft models in combination with chemotherapy (Prewett *et al*, 2004).

The dose and treatment schedule used for necitumumab in the current study was based on safety and pharmacokinetic data from a phase I study in 60 heavily pretreated patients with advanced solid tumours (Kuenen *et al*, 2010). This study established the maximum tolerated dose of necitumumab and the recommended dose for further clinical development to be 800 mg, administered intravenously (i.v.), either weekly or every second week. The major dose-limiting toxicity was grade 3 headache. The most common drug-related adverse events (AEs) were typical for this class of agent and consisted predominantly of skin reactions, headache, nausea/vomiting, and fatigue (mostly grade 1 or 2). Importantly, no hypersensitivity or infusion reactions associated with necitumumab were reported in this trial.

The present phase II study was designed to investigate necitumumab in combination with a modified version of the FOLFOX6 regimen (mFOLFOX6) and was initiated following preliminary reports (later reported in full) of encouraging activity and safety associated with cetuximab in combination with oxaliplatin-based chemotherapy (Tabernero *et al*, 2007; Boccia *et al*, 2010). Evidence at that time from small single-arm studies in chemorefractory mCRC suggested that responses to treatment with cetuximab were confined to patients whose tumours did not harbour *KRAS* codon 12 or 13 (exon 2) mutations (Di Fiore *et al*, 2007; De Roock *et al*, 2008; Lievre *et al*, 2008). These findings were later confirmed in larger randomised studies conducted in the first-line setting (Bokemeyer *et al*, 2011; Van Cutsem *et al*, 2011; Douillard *et al*, 2014). Accordingly, in March 2008, the protocol for the present study was amended to include evaluation of tumour mutation status at *KRAS* codons 12 and 13 among enrolled patients.

## Materials and methods

### Patients

Eligible patients were ⩾18 years old, with histologically confirmed locally advanced unresectable or metastatic adenocarcinoma of the colon or rectum, life expectancy ⩾6 months, and an Eastern Cooperative Oncology Group performance status (ECOG PS) of ⩽2. Patients were also required to have an EGFR-detectable or EGFR-undetectable tumour (the option of tumour biopsy was offered to patients without sufficient archived tumour tissue to allow assessment of EGFR) and at least one unidimensionally measurable target lesion (⩾2 cm with conventional techniques or ⩾1 cm with spiral computed tomography (CT) scan). Adequate haematological, hepatic, and renal function and recovery from the effects of prior therapy were also required. Key patient exclusion criteria included: prior systemic chemotherapy for locally advanced unresectable CRC or mCRC (prior adjuvant chemotherapy was allowed if progressive disease (PD) was documented >6 months after the end of the last cycle of adjuvant chemotherapy or ⩾12 months for oxaliplatin-containing regimens); prior radiotherapy to >25% of bone marrow (radiation therapy as a part of standard adjuvant chemoradiotherapy for rectal cancer >6 months before study entry was allowed); documented and/or symptomatic brain metastases; previous therapy with mAbs or any EGFR-targeting agent; current use of chronic non-topical corticosteroid treatment for >6 months at doses >10 mg per day of prednisolone or equivalent before study entry, which in the opinion of the investigator could compromise the patient or the study; known dihydropyrimidine dehydrogenase deficiency; or acute or subacute intestinal occlusion.

The study was conducted in accordance with the ethical principles of the Declaration of Helsinki and the International Conference on Harmonisation and Good Clinical Practice. Study procedures were approved by local ethic committees, and all patients provided written informed consent.

### Study design and treatment

This was an open-label, single-arm, multicentre, phase II study investigating the efficacy and safety of necitumumab in combination with mFOLFOX6 in the first-line treatment of locally advanced CRC or mCRC. A treatment cycle was defined as 2 weeks. On day 1 of each cycle, patients received necitumumab at an absolute dose of 800 mg, by i.v. infusion over 50 min. The necitumumab infusion was followed by administration of the mFOLFOX6 regimen (85 mg m^−2^ i.v. oxaliplatin over 2 h; 400 mg m^−2^ i.v. folinic acid over 2 h; and then 5-FU, 400 mg m^−2^ i.v. bolus injection followed by 2400 mg m^−2^ continuous i.v. infusion over 46 h on days 1–2). Dose modifications as specified in the study protocol were permitted in the event of treatment-related toxicity. Radiographic evaluation (CT or magnetic resonance imaging) of disease was performed every 8 weeks; treatment continued until documentation of PD, development of unacceptable toxicity, protocol noncompliance, or withdrawal of consent. For patients who discontinued treatment for reasons other than PD, radiographic evaluation of disease continued at least every 3 months after discontinuation (until PD).

### Endpoints and assessments

The primary endpoint was ORR, based on best response determined by investigators according to the Response Evaluation Criteria in Solid Tumors, Version 1.0 (Therasse *et al*, 2000). No central radiological review was carried out. Responses (complete or partial) were to be confirmed at least 4 weeks after the criteria for response were first met.

Secondary endpoints included OS, PFS, duration of response, pharmacokinetics, and safety. In addition, the association between clinical outcome (ORR, OS, and PFS) following treatment and tumour *KRAS* exon 2 mutation status (wild type *vs* mutant) and *EGFR* mutation status and EGFR protein expression status (positive *vs* negative) was evaluated.

Patient safety was evaluated at every treatment visit, based on reported AEs, serious AEs, physical examinations, and laboratory analyses. Adverse events were classified by type, incidence, severity, and causality. The National Cancer Institute–Common Terminology Criteria for Adverse Events, version 3.0, was used to grade all systemic and local AEs; all AE terms were coded using the Medical Dictionary for Regulatory Activities (MedDRA 13.0).

Tumour *KRAS* exon 2 and *EGFR* mutation screening and EGFR expression analysis were performed by Genzyme Genetics (Los Angeles, CA, USA). Mutation detection was carried out on DNA extracted from microdissected tumour sections prepared from formalin-fixed paraffin-embedded (FFPE) archival samples (following pathologist review) using an Arcturus PicoPure DNA extraction kit (Applied Biosystems, Grand Island, NY, USA). *KRAS* mutations (codons 12 and 13) were detected by PCR using the TheraScreen K-RAS Mutation Kit (Qiagen, Hilden, Germany). Epidermal growth factor receptor kinase domain mutation screening (*EGFR* exons 18–21) was carried out by PCR and bi-directional sequencing (BigDye v1.1, Applied Biosystems) on a 3130 Genetic Analyzer (Applied Biosystems). Tumour EGFR expression was detected by immunohistochemistry (IHC) performed on tissue sections prepared from FFPE samples using the DAKO EGFR pharmDx IHC kit (Glostrup, Denmark). Immunostained sections were evaluated by a pathologist according to manufacturer's guidelines.

Pharmacokinetic parameters (*C*_max_, *T*_max_, AUC_(0–tlast)_, AUC_(0–∞)_, AUC_(tlast–∞)_, *t*_½_, CL, and *V*_ss_) were calculated using non-compartmental methods (WinNonlin 5.3, Pharsight Corporation, Cary, NC, USA) from serum concentrations over time following a single dose of 800 mg necitumumab co-administered with mFOLFOX6 on day 1 of cycle 1. Blood samples were collected before and immediately after the initial necitumumab infusion, and at 1, 2, 4, 24 (day 2), 72 (day 4), 96 (day 5), 144 (day 7), 168 (day 8), and 236 (day 11) hours after infusion. From cycles 2–6, additional blood samples were drawn immediately before and 1 h after the end of necitumumab infusion. Samples were also collected at the end of therapy and 45 days after the final necitumumab infusion.

### Statistical analysis and considerations

Primary and secondary efficacy endpoints were evaluated in the modified intention to treat (mITT) population, comprising all patients who were enrolled and treated with any quantity of necitumumab or chemotherapy.

The study sample size was calculated using expected response rates of 55% for necitumumab plus mFOLFOX6 and 32% for mFOLFOX6 alone, based on a previously reported response rate of 32% for FOLFOX (Venook *et al*, 2006). Using a two-sided 95% confidence interval (CI), it was estimated that enrolment of 40 evaluable patients would give a power of 86% to detect a significant difference in ORR between necitumumab plus mFOLFOX6 and the historical control.

The Kaplan–Meier method was used to estimate time to event data (OS, PFS, and duration of response) with 95% CIs. Overall survival was defined as the time from the first day of therapy to the date of death; if the patient was alive at the end of the follow-up period or was lost to follow-up, OS was censored on the last date the patient was known to be alive. Progression-free survival was defined as the time from the first day of therapy until the date of PD or death, whichever was first. In the case where study treatment results in tumour regression allowing for surgical resection, the PFS analysis used the date of radiological progression determined by the regular follow-up radiological assessments following the surgical resection. If no progression was observed the patient was censored at the last radiological assessment. Duration of response was defined as the time from the day the measurement criteria were met for a complete or partial response (whichever was first recorded) until the first date of PD or death. Patients who neither experienced PD nor died were censored at the date of their last tumour assessment.

Exploratory subgroup analyses of ORR, OS, and PFS by tumour *KRAS* exon 2 mutation status and EGFR expression status were performed. The evaluation based on *KRAS* exon 2 mutation status was not planned in the original study protocol (6 February 2007), but was added in a protocol amendment (17 March 2008) for tumour tissue samples submitted by patients who signed an informed consent document specific to this evaluation. No other substantive changes to the conduct of the study were made.

The database was locked for analysis on 31 March 2011. Data analyses were performed using SAS 8.2 or higher.

## Results

### Patients and treatment

A total of 44 patients were enrolled between 01 August 2007 and 03 June 2008, at three sites in Spain and two in Belgium; all 44 enrolled patients received at least one dose of study therapy; the mITT population may therefore be considered to be equivalent to a classical ITT population. All patients discontinued study treatment, mostly due to PD (*n*=18, 40.9%) or AEs (*n*=10, 22.7%). Patient baseline and disease characteristics are summarised in Table 1.

All patients had a diagnosis of adenocarcinoma, originating from the colon (65.9%) or rectum (34.1%), and either metastatic (95.5%) or locally advanced (two patients; 4.5%) disease at baseline. The mean duration of disease (time from initial diagnosis to the date of first dose of any study treatment) was 5.1 months (range: 0.3–42.8 months). The majority of patients (*n*=41; 93.2%) had a baseline ECOG PS of 0–1.

Of the 44 enrolled patients, 33 (75.0%) received ⩾80% of the planned doses of necitumumab, oxaliplatin, and folinic acid; 26 patients (59.1%) received ⩾80% of the planned dose of 5-FU (Supplementary Table 1). The number of treatment delays of at least 1 week was similar across all agents. No dose reductions were required for necitumumab; 11.4% and 31.8% of patients required ⩾2 reductions to the dose of oxaliplatin and 5-FU, respectively.

### Efficacy

All 44 patients in the ITT population were evaluable for the best overall response. The ORR was 63.6% (95% CI 47.8–77.6), including four patients (9.1%) with a complete response and 24 (54.5%) with a partial response (Table 2). An additional 15 patients (34.1%) had stable disease as best response, resulting in a disease control rate of 97.7% (95% CI 88.0–99.9). The median duration of response was 10.0 months (95% CI 7.0–16.0).

**Figure 1:**
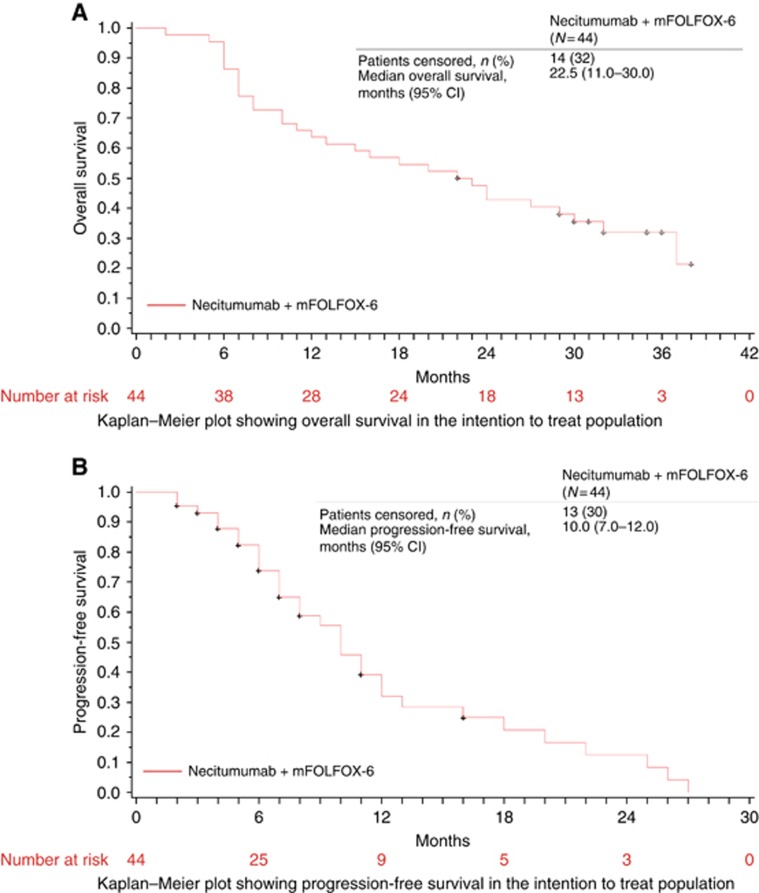
**Overall survival and progression free survival data in the ITT population.** Kaplan–Meier plots showing overall survival (**A**) and progression-free survival (**B**) in the intention to treat population.

A total of 30 deaths were reported; 13 patients were alive at the cut-off date (31 October 2010), and one was lost to follow-up. The median OS was 22.5 months (95% CI 11.0–30.0). At 1- and 2-year OS, the rates were 63.6% and 42.9%, respectively (Figure 1). In the analysis of PFS, 31 events (documented PD or death) were observed and 13 patients were censored (Figure 1); the median PFS was 10.0 months (95% CI 7.0–12.0).

*KRAS* exon 2 tumour mutation status was evaluable in tumours from 25 of the 44 enrolled patients (results were not available for 19 patients, either because adequate tumour tissue was not available or the patient did not provide consent to the additional analysis). *KRAS* codon 12 or 13 mutations were detected in the tumours from 9 of these 25 patients (36.0%), and the tumours from the remaining 16 patients (64.0%) were wild type at these loci.

**Figure 2:**
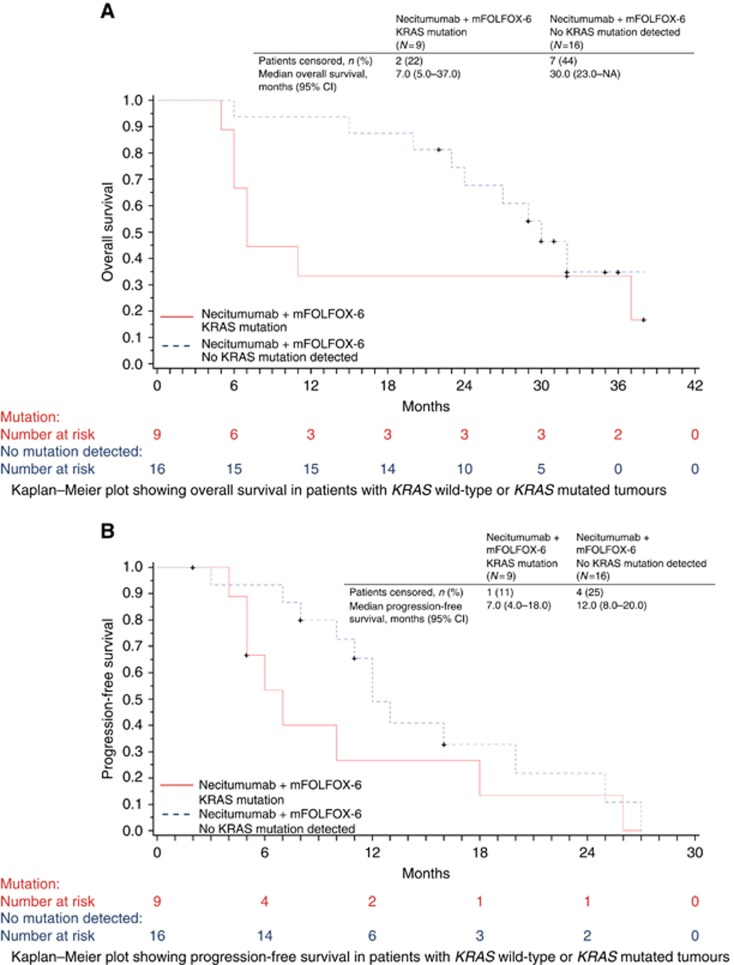
**Overall survival and progression free survival data in the ITT population.** Kaplan–Meier plots showing overall survival (**A**) and progression-free survival (**B**) in patients with *KRAS* wild-type or *KRAS* mutated tumours.

The ORR was higher in patients with *KRAS* exon 2 wild-type tumours (87.5%, 95% CI 61.7–98.4) compared with those with *KRAS* exon 2 mutated tumours (55.6%, 95% CI 21.2–86.3), with all four complete responses reported in the study occurring in patients with *KRAS* exon 2 wild-type tumours. Overall survival (median 30.0 months (95% CI 23–NA) *vs* 7.0 months (95% CI 5.0–37.0)) and PFS (median 12.0 months (95% CI 8.0–20.0) *vs* 7.0 months (95% CI 4.0–18.0)) were also longer among patients with *KRAS* exon 2 wild-type tumours compared with those with *KRAS* exon 2 mutant tumours (Figure 2).

No EGFR kinase domain mutations were detected in any of the tumours from 36 patients evaluable for *EGFR* tumour mutation status.

Tumour EGFR protein expression status was evaluable for 37 of the 44 enrolled patients; data were missing for five patients, and the analysis was not performed for two patients. A total of 16 (43.2%) of 37 patients had tumours that were positive for EGFR expression by IHC; 21 (56.8%) had tumours that were EGFR negative. Overall, there were no significant differences between these subgroups for any of the efficacy parameters evaluated. The ORR was 62.5% (95% CI 35.4–84.8) among patients with EGFR-expressing tumours and 66.7% (95% CI 43.0–85.4) among those whose tumours were negative for EGFR expression. Median OS (25.5 months *vs* 24.0 months) and median PFS (11.0 months *vs* 10.0 months) were also similar between these subgroups.

### Safety

Treatment-emergent AEs of any grade were reported in all treated patients. Grade ⩾3 events were reported in 38 (86.4%) patients; the most common of these events were neutropenia (29.5%), asthenia (27.3%), and rash (20.5%) (Table 3). Grade 4 AEs were reported for seven patients (15.9%). Six of these seven patients had grade 4 AEs considered to be related to study treatment by the investigator, including four patients with grade 4 neutropenia and one patient each with sepsis and febrile neutropenia. In addition, one patient was reported with grade 4 hypocalcaemia (considered to be not related to study treatment).

Consistent with prior evidence that skin toxicity is a class effect of EGFR mAbs, 41 patients (93.2%) experienced some form of skin or subcutaneous disorder regardless of grade or relationship to study therapy. This included five patients (11.4%) with acneform dermatitis and 31 patients (70.5%) with rash. Skin toxicity was grade 3 in a total of 13 patients (29.5%), including 1 of 5 patients with acneform dermatitis (2.3%) and 9 of 31 with rash (20.5%). In all patients, rash and acneform dermatitis were considered at least possibly related to necitumumab. Conjunctivitis was observed in 11 patients (25.0%), and was grade 3 in one (2.3%); 9 of 11 patients had conjunctivitis considered at least possibly related to necitumumab (including the patient with grade 3 conjunctivitis). In addition, 10 patients (22.7%) experienced palmar-plantar erythrodysesthesia syndrome (including two patients (4.5%) of grade 3); necitumumab-related palmar-plantar erythrodysesthesia syndrome was reported in 7 (15.9%) patients (including 1 (2.3%) with grade 3).

Regarding AEs of special interest, hypersensitivity reactions were reported in three patients (6.8%); these included one serious grade 2 AE that was considered to be related to necitumumab, and one grade 1 and one grade 2 AE that were considered to be related to chemotherapy. Conjunctivitis of any grade was reported in 11 patients (25.0%); events were considered to be related to necitumumab for nine of these patients, including one with a grade 3 event. Hypomagnesaemia was reported in five patients overall; three patients experienced grade ⩽2 hypomagnesaemia considered to be related to necitumumab. Thromboembolic AEs were reported in nine patients (20.5%), including six patients with grade 2 events and three patients with grade 3 events. Of these AEs, only one (grade 2 thrombosis in device) was considered to be at least possibly related to necitumumab.

Adverse events commonly associated with exposure to oxaliplatin-based chemotherapy included: neutropenia (52.3% grade ⩾3, 29.5%), thrombocytopenia 11.4% (all grade 1–2), and peripheral sensory neuropathy (22.7% grade 3, 9.1%). All of these AEs were considered to be at least possibly related to study therapy.

Treatment discontinuations due to AEs (primary cause) were reported in 10 (22.7%) patients; these included one patient each with grade 1 thrombocytopenia, persistent grade 2 mucositis, grade 2 asthenia (worsening) with grade 2 neuropathy, grade 2 paraesthesia, grade 3 dizziness, grade 3 vomiting, grade 3 fatigue, and grade 3 asthenia. This group also included two patients who died due to an AE (grade 5 event), one from respiratory failure and one from intestinal obstruction. In total, there were three grade 5 treatment-emergent AEs in this study, including one additional patient with respiratory failure (including organising pneumonia). Two additional deaths, one due to intestinal obstruction and one due to respiratory failure secondary to pneumonia, were not reported as treatment-emergent AEs as they occurred >30 days after the last dose of study therapy. Twenty-five additional deaths occurred >30 days after the last dose of study therapy due to PD, including one patient whose death was deemed to be due to general health deterioration, which was considered secondary to PD. None of the reported deaths were considered to be related to any study therapy.

### Pharmacokinetics

Following infusion of 800 mg necitumumab on day 1 of cycle 1, at least one sample for pharmacokinetic analysis was collected from 43 of 44 enrolled patients, with data available from 42 (95.5%) patients. Data from three patients were excluded due to high pre-dose concentrations or limited available data points. Summary data for the non-compartmental analysis of the necitumumab pharmacokinetic parameters are shown in Supplementary Table 2. The geometric mean *C*_max_ was 344 *μ*g ml^−l^ (coefficient of variation (CV)=46%) following single-dose administration. The geometric means of estimated half-life and clearance were 142 h (range 99.8–299) and 20.3 ml h^−l^ (CV=35%), respectively. Following multiple-dose administration (800 mg every 2 weeks), geometric mean (CV%) trough levels appeared to reach a plateau after 4–5 cycles, ranging from 59.1 (60) to 75.4 (64) *μ*g ml^−1^ (Supplementary Figure 1). Data from a pre-clinical study indicated that necitumumab was effective at or above trough concentrations of 40 *μ*g ml^−1^. The median trough concentration observed in the current study exceeded this target level from cycle 3.

## Discussion

This phase II study was designed to evaluate the efficacy and safety of the second-generation human EGFR mAb necitumumab in combination with mFOLFOX6 in previously untreated patients with locally advanced or metastatic CRC. The study was initiated at a time when other EGFR mAbs (cetuximab and panitumumab) were also in clinical development.

The majority of the 44 treated patients (95.5%) had metastatic disease; baseline demographic and disease characteristics were generally comparable with those reported for other studies investigating chemotherapy in combination with targeted agents in previously untreated patients with mCRC (Tabernero *et al*, 2007; Hochster *et al*, 2008; Bokemeyer *et al*, 2009). Pharmacokinetic analysis of necitumumab largely confirmed the findings reported from the phase I study of necitumumab in patients with solid malignancies (Kuenen *et al*, 2010), with target trough concentrations (⩾40 *μ*g ml^−l^) achieved in most patients after three cycles of treatment. The data suggest that co-administration of mFOLFOX6 had no relevant impact on the pharmacokinetic characteristics of necitumumab.

In the ITT population, the primary endpoint of ORR was 63.6% (95% CI 47.8–77.6); this compared favourably with the ORR of 41% (95% CI 27–56) measured in unselected patients with mCRC receiving mFOLFOX6 alone in the randomised phase III TREE study (Hochster *et al*, 2008). Similarly, OS in the present study compared favourably (median 22.5 months, 95% CI 11.0–30.0) with that reported in the TREE study (19.2 months, 95% CI 14.2–24.9).

Response rates in the current study were also comparable with those reported in studies of cetuximab in combination with FOLFOX regimens (44.8–72%) in unselected patients with mCRC (Tabernero *et al*, 2007; Bokemeyer *et al*, 2009; Boccia *et al*, 2010; Colucci *et al*, 2010). In addition, the secondary endpoints of OS and PFS in the current study were also comparable with those reported from these previous studies in this setting.

The effect of tumour mutations in *KRAS* codons 12 or 13 on the efficacy of necitumumab in combination with mFOLFOX6 was studied in 25 evaluable patients. The number of patients studied was small, and the findings should be treated with caution. However, there was a tendency towards a higher ORR (87.5% *vs* 55.6%), longer PFS (median 12.0 *vs* 7.0 months), and longer OS (median 30.0 *vs* 7.0 months) among patients whose tumours were *KRAS* exon 2 wild type compared with those whose tumours harboured *KRAS* exon 2 mutations. These findings support those from other single-arm studies of EGFR mAbs in previously treated (Di Fiore *et al*, 2007; De Roock *et al*, 2008; Lievre *et al*, 2008) and untreated patients (Colucci *et al*, 2010), which were later confirmed in larger randomised studies of first-line FOLFOX4 in combination with either cetuximab or panitumumab (Bokemeyer *et al*, 2011; Douillard *et al*, 2014). Further investigation is required to assess whether mutations at other *RAS* loci are negative predictors of efficacy for necitumumab in combination with a FOLFOX regimen, as has been reported for cetuximab and panitumumab (Douillard *et al*, 2013; Bokemeyer *et al*, 2015).

In further subgroup analyses, no relevant differences were reported for any of the studied efficacy endpoints in patients whose tumours were positive for EGFR expression compared with those whose tumours were negative. Thus, it appears that EGFR expression as detected by IHC is not a requirement for the efficacy of necitumumab in combination with chemotherapy, consistent with findings from other studies evaluating cetuximab (Chung *et al*, 2005; Folprecht *et al*, 2010; Brodowicz *et al*, 2013; Licitra *et al*, 2013).

The safety profile of necitumumab in combination with mFOLFOX6 was generally comparable with those reported for other EGFR mAbs when used in combination with FOLFOX regimens (Tabernero *et al*, 2007; Folprecht *et al*, 2010; Ocvirk *et al*, 2010; Bokemeyer *et al*, 2011; Brodowicz *et al*, 2013; Douillard *et al*, 2014; Wasan *et al*, 2014). The most common grade ⩾3 AEs were primarily class effects reflecting exposure to this treatment combination, including gastrointestinal disorders (diarrhoea 9.1%), blood and lymphatic disorders (neutropenia, 29.5%), nervous system disorders (paraesthesia 13.6%), and skin toxicities (rash 20.5%). In line with expectations for a human antibody, the incidence of hypersensitivity reactions was low: reported in only three patients (one at grade 1, two at grade 2), the grade 2 reaction in one patient was considered to be related to necitumumab. Notably, venous thromboembolic events have previously been associated with the administration of EGFR antibodies in combination with chemotherapy (Petrelli *et al*, 2012). Of the eight thromboembolic events reported in the current study, none were grade >3 and only one was considered to be possibly related to necitumumab. There were no reports of cardiac ischaemia. Of note, the incidence of grade ⩾3 asthenia (27.3%) was somewhat higher than generally reported in other studies of EGFR mAbs in combination with FOLFOX regimens. Adverse events leading to treatment discontinuation were reported in 10 (22.7%) patients and none of the 30 reported deaths were considered to be treatment related.

In summary, the combination of necitumumab with mFOLFOX6 was associated with evidence of efficacy and a manageable safety profile in patients with previously untreated locally advanced or metastatic CRC. Clinical outcome was better in patients with *KRAS* exon 2 wild type compared with *KRAS* exon 2 mutant tumours. The efficacy of this combination appears to be at least comparable with other EGFR antibodies approved for use in combination with first-line oxaliplatin-based chemotherapy in patients with mCRC. Although cross-trial comparisons should be treated with caution, the data suggest that combining necitumumab with mFOLFOX6 has the potential to provide additional benefit in this setting. Further investigation of necitumumab with oxaliplatin-based chemotherapy in patients with tumours wild type for *RAS* is required if this EGFR mAb is to be added to the therapeutic armamentarium of mCRC.

## Figures and Tables

**Table 1:** Baseline characteristics in the ITT population

**Characteristic**	***N*****=44**
**Sex**	
Male	25 (56.8)
Female	19 (43.2)
**Race**	
White	42 (95.5)
Black	2 (4.5)
**Age group**	
18 to <65	22 (50.0)
⩾65	22 (50.0)
Median age, years (range)	64.0 (33–81)
**Duration of disease (months)**	
Mean (s.d.)	5.1 (9.8)
Median (range)	1.6 (0.3–42.8)
**ECOG performance status**	
0	22 (50.0)
1	19 (43.2)
2	3 (6.8)
**Disease**	
Locally advanced	2 (4.5)
Metastatic	42 (95.5)
**Site of tumour origin**	
Colon	29 (65.9)
Rectum	15 (34.1)
**Tumour grade**	
Well differentiated	8 (18.2)
Moderately differentiated	23 (52.3)
Poorly differentiated	4 (9.1)
Undifferentiated	0
Unknown	9 (20.5)
**Tumour EGFR expression status (IHC)**	
Expressing	16 (36.4)
Negative	21 (47.7)
NA	2 (4.5)
Missing	5 (11.4)

Abbreviations: ECOG=Eastern Cooperative Oncology Group; IHC=immunohistochemistry; ITT=intention to treat; NA=not available.

Data presented are *n* (%) unless otherwise stated.

**Table 2:** Disease response in the ITT population and in patients grouped by tumour KRAS mutation status

		***KRAS*** **evaluable patients (*****N*****=25)**^a^	
**Parameter**	**ITT (*****N*****=44)**	***KRAS*** **wild type (*****n*****=16)**	***KRAS*** **mutant (*****n*****=9)**
**Best overall response,** ***n*** **(%)**			
Complete response	4 (9.1)	4 (25.0)	0
Partial response	24 (54.5)	10 (62.5)	5 (55.6)
Stable disease	15 (34.1)	2 (12.5)	4 (44.4)
Progressive disease	1 (2.3)	0	0
Not evaluable	0	0	0
ORR, *n* (%)	28 (63.6)	14 (87.5)	5 (55.6)
95% CI	47.8–77.6	61.7–98.4	21.2–86.3
DCR, *n* (%)	43 (97.7)	16 (100)	9 (100)
95% CI	88.0–99.9	79.4–100	66.4–100

Abbreviations: CI=confidence interval; DCR=disease control rate; ITT=intention to treat; ORR=objective response rate.

a Missing in 19 patients.

**Table 3:** Treatment-emergent adverse events in the ITT population (*N*=44)

**Adverse event**^a^	**Any grade**	**Grade** ⩾**3**
Any	44 (100)	38 (86.4)
**Blood and lymphatic disorders**		
Neutropenia	23 (52.3)	13 (29.5)
Anaemia	9 (20.5)	1 (2.3)
Thrombocytopenia	5 (11.4)	0
**Eye disorders**		
Conjunctivitis	11 (25.0)	1 (2.3)
**Gastrointestinal disorders**		
Diarrhoea	24 (54.5)	4 (9.1)
Nausea	17 (38.6)	1 (2.3)
Vomiting	16 (36.4)	3 (6.8)
Constipation	13 (29.5)	1 (2.3)
Stomatitis	8 (18.2)	0
Abdominal pain upper	5 (11.4)	0
Dyspepsia	5 (11.4)	0
Internal obstruction	4 (9.1)	4 (9.1)
**General disorders and administration site conditions**		
Asthenia	36 (81.8)	12 (27.3)
Mucosal inflammation	19 (43.2)	2 (4.5)
Pyrexia	10 (22.7)	0
**Infections and infestations**		
Paronychia	16 (36.4)	1 (2.3)
**Investigations**		
Weight decreased	14 (31.8)	0
Weight increased	7 (15.9)	0
**Metabolism and nutrition disorders**		
Decreased appetite	18 (40.9)	2 (4.5)
Hypomagnesaemia	5 (11.4)	0
**Musculoskeletal and connective tissue disorders**		
Back pain	8 (18.2)	1 (2.3)
**Nervous system disorders**		
Paraesthesia	16 (36.4)	6 (13.6)
Dysaesthesia	13 (29.5)	4 (9.1)
Peripheral sensory neuropathy	10 (22.7)	4 (9.1)
Neurotoxicity	9 (20.5)	1 (2.3)
Dysgeusia	7 (15.9)	0
**Respiratory, thoracic, and mediastinal disorders**		
Dyspnoea	5 (11.4)	0
**Skin and subcutaneous tissue disorders**		
Rash	31 (70.5)	9 (20.5)
Alopecia	10 (22.7)	0
Palmar-plantar erythrodysaesthesia syndrome	10 (22.7)	2 (4.5)
Skin fissures	10 (22.7)	2 (4.5)
Dry skin	8 (18.2)	0
Dermatitis acneiform	5 (11.4)	1 (2.3)
Pruritus	5 (11.4)	0

Abbreviation: ITT=intention to treat.

a Data presented are *n* (%) according to preferred terms in order of system organ class coded by Medical Dictionary for Regulatory Activities for any grade occurring in ⩾10% of patients or for grade ⩾3 occurring in ⩾5% of patients.
