# Supplementary figures and images for: Phase II study of necitumumab plus modified FOLFOX6 as first-line treatment in patients with locally advanced or metastatic colorectal cancer
Source: Br J Cancer. 2016 Jan 14;114(4):372–80. doi: 10.1038/bjc.2015.480 (PMC4815776; doi:10.1038/bjc.2015.480)

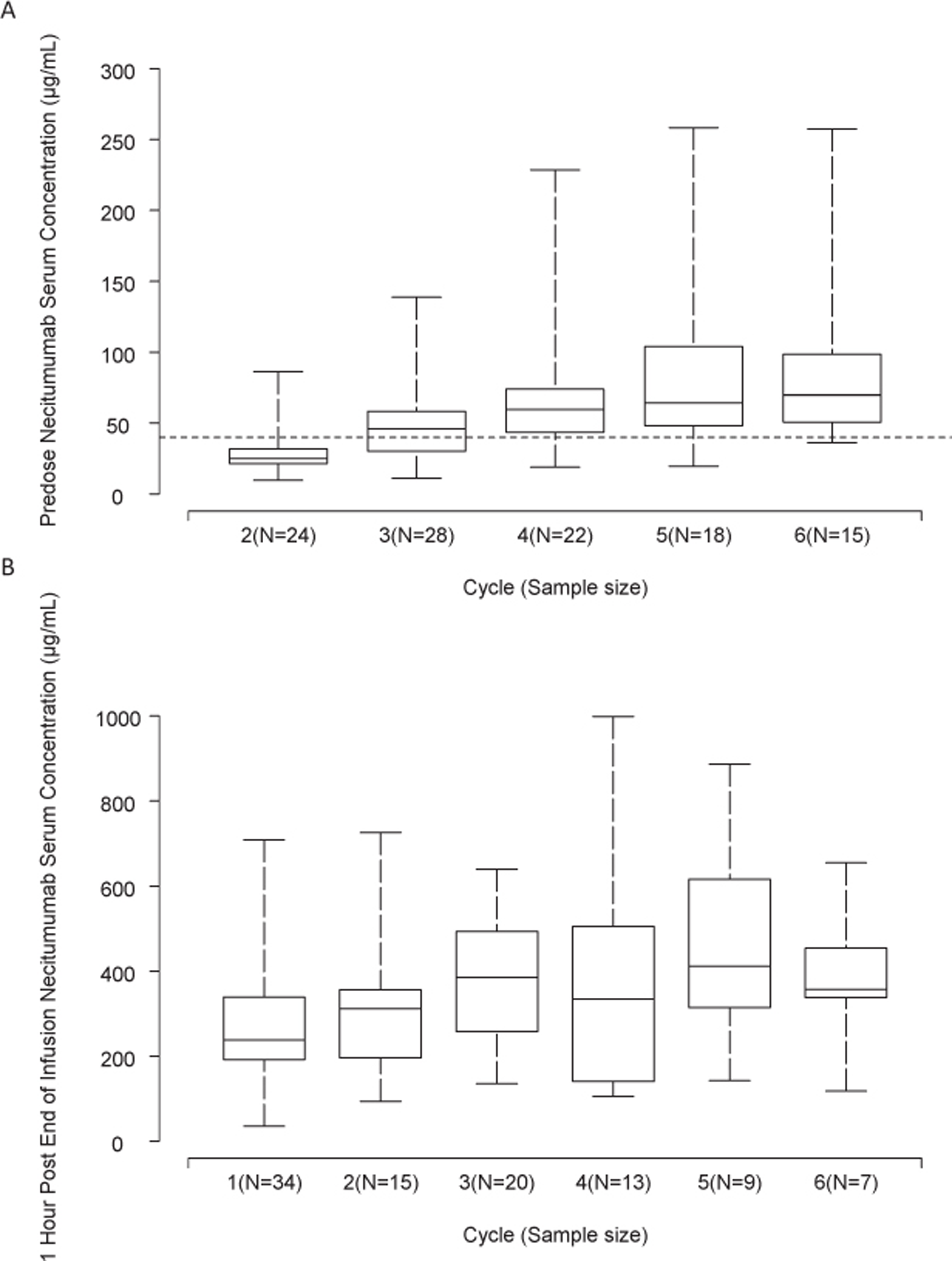

Supplement: Supplementary Figure 1 [file bjc2015480x1.tif]
