# Supplementary material for: Phase II study of necitumumab plus modified FOLFOX6 as first-line treatment in patients with locally advanced or metastatic colorectal cancer
Source: Br J Cancer. 2016 Jan 14;114(4):372–80. doi: 10.1038/bjc.2015.480 (PMC4815776; doi:10.1038/bjc.2015.480)
Supplement: Supplementary Figure Legends [file bjc2015480x2.docx]

**Supplementary Figure 1.** Necitumumab serum concentration-time profiles, following multiple doses of 800 mg on an every-2-Week Dosing Regimen (*N*=43)

A) predose and B) 1 hr post end-of-infusion.

The middle line in each box plot represents the median; the top and bottom margins of the box represent the 75th and 25th percentiles; the whiskers extend to the maximum and minimum values. The dashed line represents 40 μg/mL.
